# Participating in Different Activities and Their Association with Mental Health Problems in the Working Disabled Population in Korea

**DOI:** 10.3390/ijerph19148348

**Published:** 2022-07-08

**Authors:** Doukyoung Chon, Jong Youn Moon, Jae-Hyun Kim

**Affiliations:** 1 Center for Public Health, Gil Medical Center, Gachon University College of Medicine, Incheon 21936, Korea; cdytgtg@naver.com; 2 Institute for Digital Life Convergence, Dankook University, Yongin 16890, Korea; 3 Department of Preventive Medicine, Gachon University College of Medicine, Incheon 21936, Korea; 4 Artificial Intelligence and Big-Data Convergence Center, Gil Medical Center, Gachon University College of Medicine, Incheon 21936, Korea; 5 Department of Public Health Administration, Dankook University, Yongin 16890, Korea

**Keywords:** disabled, social activity, leisure activity, stress, depression

## Abstract

Background: There are a large number of people suffering from disabilities and this number is rapidly rising. People with disabilities experience various hardships and are more vulnerable to mental health problems. Participating in different types of activities (e.g., leisure, social, etc.) has been shown to ameliorate people’s mental health problems such as stress and depression. The aim of this study was to assess the effects of leisure and social activities on the mental well-being of the working disabled population in Korea. Methods: A total of 1521 disabled people aged between 15 and 64 were included in the study. The degree of participation in leisure (i.e., weekends or weekdays) and social activities, as well as stress and depressive symptoms, were measured using single-item questions. The association between participation in different activities (i.e., leisure and social) and mental health (i.e., stress and depression) was analyzed using a generalized estimating equation (GEE) model. Results: In the fully adjusted model, participation in leisure activities was associated with the level of stress, and less leisure participation was significantly associated with higher odds of stress. For example, in the “2 or fewer hours” group (odds ratio [OR] = 1.461, 95% confidence interval [CI] = 1.193–1.789) with the “5 h or more” group used as the reference for weekdays and the “5–9 h” group (OR = 1.223, 95% CI = 1.007–1.486) with the “10 h or more” group used as the reference for weekends. In terms of participation in social activities, increased participation was associated with lower levels of depression. For example, in the “Very much” group (OR = 0.314, 95% CI = 0.156–0.633) compared to the “Not at all” group. Conclusions: Participation in different activities was associated with better mental health outcomes in the working disabled population in Korea.

## 1. Introduction

Disability is a growing public health concern, as more than 1 billion people are living with a disability and this number is rapidly increasing [1]. Furthermore, the number of working disabled people is growing and in 2018 there were 13,564 (i.e., approximately 3.2% of the total working population) [2] working disabled people, accentuating the need to research the well-being of this population. People with disabilities experience various hardships in their lives including functional limitations as well as social stigmatization [3]. Moreover, these difficulties not only affect the daily lives of the disabled population but also increase their vulnerability to hazardous outcomes such as poor mental health (i.e., stress, depression) [4,5]. Disability, stress, and depression appear to be intertwined with one another and people with disabilities experience stress due to their limited ability in certain aspects of life [6], which could then negatively affect their depressive symptomatology. Furthermore, the presence of depressive symptoms could worsen an individual’s disability [7], creating a cycle of association between the three factors. Accordingly, investigating the factors that could ameliorate this deteriorating cycle would benefit disabled people’s well-being. One factor that could be considered in this regard is participation in different types of activities [8,9].

Leisure activities are activities that individuals voluntarily participate in because they find them enjoyable, and participating in different types of leisure activities is associated with lower negative mental health outcomes in various populations [10]. For instance, Pressman et al. [8] included four different samples of participants in their study and found an association between relaxing leisure activities and lower levels of depression. Additionally, Chang and Yu [11] selected a more detailed aspect of leisure (i.e., leisure autonomy, leisure competency, and leisure social support), and all three categories were negatively associated with stress. Social activities have also shown a positive effect on mental health. The beneficial effects of participating in social activities have been presented in various studies [9,12,13]. Furthermore, Hong, Hasche, and Bowland [14] analyzed eight different categories of social activity in an effort to more clearly understand the association between participating in social activities and depression. These studies not only demonstrate the importance of participating in social activities but the necessity to regard this factor in different settings.

Despite available research on the association between disability and mental health as well as the benefits of participating in different activities and mental health, studies considering the factors together are scarce. However, considering the demonstrated positive effects of participating in different activities for other populations, we hypothesized that participating in those activities could also benefit the disabled population. The aim of this study was to assess the effects of leisure and social activities on the mental well-being of the working disabled population in Korea. Including different types of activities in association with mental health (i.e., stress, depression) could provide valuable indications as to the types of activities that most benefit different mental health conditions.

## 2. Materials and Methods

### 2.1. Study Sample

Data used in the study were obtained from the second wave of the Panel Survey of Employment for the Disabled (PSED), which is a longitudinal study of Koreans registered as disabled between the ages of 15 and 64. PSED was designed to collect valuable data providing time-series continuity in various reported topics. The first survey was conducted on 15 May 2016, accounting for factors such as region, disability type, disability grade, etc., to achieve a nationally representative sample.

The first survey in 2016 interviewed 4577 participants with questions related to basic characteristics, economic activity, daily life, quality of life, etc., using Table PC-Assisted Personal Interviewing (TAPI). PSED intended to reinterview the same participants annually. In the second year, the interviewers checked the participants’ responses from the previous year and then inquired about any changes in the past year. The retention rates of the follow-up surveys were 92.1%, 4214 participants, in the second survey, and 89.7%, 4104 participants, in the third survey. Of the 4104 participants in the third survey, after excluding participants with missing values for the variables of interest, 1521 participants were included in the current study.

### 2.2. Independent Variables

Degree of participation in different activities was considered in two circumstances (i.e., participation in social activities in daily life or daily average leisure time). Degree of participation in social activities was measured by the participants’ responses to “Degree of participation in social activities in daily life”. Possible responses were “Not at all”, “Not so much”, “Somewhat”, and “Very much”. Daily average leisure time was considered in two different circumstances (i.e., weekends or weekdays). The responses to this query were categorized into three groups for each of the circumstances. For weekends, “4 or fewer hours”, “5–9 h”, and “10 h or more”, and for weekdays, “2 or fewer hours”, “3–4 h”, and “5 h or more”. 

### 2.3. Dependent Variables

Participants’ stress and depressive symptoms were measured by single-item questions. Stress was measured by the query, “amount of stress in daily life”. Possible responses were 1: “Not at all”, 2: “Not very much”, 3: “Insignificant”, 4: “Moderate”, and 5: “High”. The responses were either categorized as “No” (1~3) or “Yes” (4~5). Depressive symptoms were based on the query, “Experience of a depressive symptom within the past year”. Possible responses were 1: “Yes” or 2: “No”, and these were used as the categorical variables for the depressive symptom. The questionnaires were developed and validated by professors and other experts in the related fields before their implementation in the survey.

### 2.4. Control Variables

Covariates used in the study included gender (male, female), age (15~29, 30~39, 40~49, 50~59, 59<), residential region (metropolitan, urban, rural), marital status (married, single, divorced, or separated), self-rated health (poor, good), smoking status (current smoker, former smoker, non-smoker), alcohol consumption (drinker, former drinker, non-drinker), year (2016, 2017, 2018), stress, depressive symptoms, disability grade, and disability type. Stress and depressive symptoms were controlled depending on the dependent variable (e.g., control for stress when depressive symptoms was set as the dependent variable). Disability grade was based on the participants’ free responses to the query “disability grade” and the reported grades were categorized into either “1–3” or “4–6”. Disability type was based on the query “disability types”, which had 15 possible responses, and was categorized into two groups (physical disability, other).

### 2.5. Analytical Approach and Statistics

The differences between the characteristics of the respondents were analyzed using the chi-square test. Considering the repeated measurement data, a generalized estimating equation (GEE) model was performed to examine the association between the degree of participation in social activities in daily life and the daily average leisure time and mental health (i.e., stress and depression) of the disabled people. GEE model was used in this study because of its ability to take the participants’ repeated responses to all variables (i.e., independent, dependent, and control) into account. *p*-value < 0.05 was considered statistically significant. All statistical analyses were performed using SAS statistical software package version 9.4 (SAS Institute Inc., Cary, NC, USA).

## 3. Results

### 3.1. General Characteristics of Participants

The general characteristics of participants are shown in Table 1. Of the 1521 participants, 56.5% responded that they had stress and 11.0% reported having depressive symptoms. With regard to the degree of participation in social activities, the group with the higher degree of social participation presented the lowest prevalence of stress (49.5%) and depressive symptoms (6.1%). In terms of daily average leisure time, the group with the lower hours of average leisure time presented a higher prevalence of stress and depressive symptoms during both weekdays (62.9%, 12.2%) and weekends (57.7%, 12.6%).

### 3.2. Association between Average Leisure Time and Mental Health

The association between average leisure time and mental health, controlling for all covariates, is presented in Table 2. On the weekdays, as the amount of leisure time decreased, the level of stress tended to increase; the “3–4 h” (OR = 1.261, 95% CI: 1.050~1.515, *p* = 0.013) and “2 or fewer hours” (OR = 1.461, 95% CI: 1.193~1.789, *p =* 0.013) groups presented significantly higher ORs compared to the “5 h or more” group. A similar trend was apparent on the weekends; the “4 or fewer hours” (OR = 1.176, 95% CI: 0.946~1.463, *p* = 0.145) and “5–9 h” (OR = 1.223, 95% CI: 1.007~1.486, *p* = 0.43) groups presented a higher level of stress compared to the “10 h or more” group, but the OR of the “4 or fewer hours” group was not statistically significant. In terms of depressive symptoms, leisure time did not present any significant results. 

### 3.3. Association between Degree of Participation in Social Activities and Mental Health

The association between the degree of participation in social activities and mental health, controlling for all covariates, is presented in Table 3. In terms of stress, the degree of participation in social activities did not present any significant associations, but in terms of depressive symptoms, an increasing degree of participation was associated with a gradient decrease in depressive symptoms. The “Not much” (OR = 0.711, 95% CI: 0.502~1.005, *p* = 0.054), “Somewhat” (0.356, 95% CI: 0.246~0.515, *p* < 0.0001), and “Very much” (OR = 0.314, 95% CI: 0.156~0.633, *p* = 0.001) groups presented a significantly reduced likelihood of reporting depressive symptoms compared to the “Not at all” group. 

## 4. Discussion

In this study, we investigated the association between different types of activities (i.e., social and leisure) and mental health (i.e., stress and depressive symptoms) in the disabled population. The results of our study presented similar findings to previous studies [15,16,17,18,19]. Our findings indicate that people who participated in different forms of activities experienced better mental health. Moreover, having more leisure time presented an association with stress, and participating in more social activities presented an association with depressive symptoms in the disabled population. Considering our findings, understanding the possible pathways of association between the variables appears to be an imperative point to consider in this line of work.

Regarding the association between leisure activity and mental health, our study focused on the utilized leisure time of the participants. According to previous studies, leisure was identified as a possible gateway to achieving relaxation [20,21] and provides assistance for coping with different stressors [10,22]. Our results were in accordance with previous studies and a shorter leisure time was associated with a greater level of stress. Considering that participating in leisure activities provides a chance to relax and reduce stress, having less time to achieve this state of relaxation appears to be the possible cause of the increased level of stress. Furthermore, the subjects in this study were working, disabled people, who have shown to experience more hardships (e.g., discrimination [23], inability to compensate for difficulties in life [24], etc.), and finding the time to relax could be a more salient factor compared to the healthy population. 

Social activities are also activities that people partake in voluntarily, but they involve more social aspects (e.g., social networks, social support, etc.), which could be the possible rationale for their association with depressive symptoms. Previous studies have shown the beneficial effects of social support and social network on depression [25]. Furthermore, a longitudinal study of Koreans found an inverse association between social activity and depression [13,26]. When the disabled population participates in social activities, they gain the chance to interact with others, which could provide social support, thus forming a wider social network. One of the benefits of participating in social activities is that disabled people can meet a wider range of people who can provide assistance in difficult situations [27] and help ameliorate depressive symptoms. Depression could lead to further problems such as impaired psychosocial functioning or poor health outcomes [28]. In this regard, examining a clear causal pathway between social activities and depression could lead to future improvements in working disabled people’s social activities, consequently improving depressive symptoms. 

Notably, considering the close association between stress and depression [29], the necessity to further investigate the variables included in the study is warranted. According to our results, leisure activities seem to have a stronger effect on stress, whereas social activities seem to have a stronger effect on depressive symptoms. This suggests that different aspects of the mental health of the disabled population are influenced by different types of activities. Furthermore, understanding the causal pathways of the variables included in this study could better assist in the improvement of mental well-being in this population.

The current study provides valuable insights regarding the mental health of the disabled population in Korea. The data used in the study was based on a nationally representative sample of the disabled population with follow-up data on the same participants and elucidated the associations between different types of activities and mental health. Furthermore, the analytical approach used in our study was useful in an effort to provide stronger statistical values between the variables.

Despite the strengths of the current study, there are some limitations that need to be considered. First, only the registered disabled population was included in the study, so the unregistered disabled population was not considered. Second, the items used in the analysis were not based on validated questionnaires but on queries, which evaluated the participants’ subjective opinions. However, previous studies have demonstrated the possible benefits of subjective measures [30,31], providing evidence to make this limitation less significant. Third, the items used to define leisure and social activities were limited. In particular, participation in leisure activities was determined by asking about the amount of leisure time, which is not sufficient for distinguishing the types of leisure activities that were exercised or for how long they were exercised. Furthermore, participants’ perceptions regarding the activities were not included in the survey. In this regard, it is necessary to use variables that can concisely measure the quality and quantity of different activities. Lastly, the research results refer only to associations rather than causal relationships and therefore need to be interpreted with caution.

In conclusion, participating in different activities was beneficial in terms of the mental health of the disabled population in Korea. Our study also provides implications for the importance of studying different types of activities and their effects on different facets of mental health. Additionally, studies from other countries [32,33,34] have reported a significant association between disabilities and other mental health disorders (e.g., anxiety, PTSD, etc.). In this regard, tailoring a mental health promotion program considering the most prominent mental health disorders in a particular country is necessary. Further research in this area could provide better assistance for the mental well-being of disabled populations around the world. 

## 5. Conclusions

According to our findings, participating in different activities was an important factor affecting the mental health of the disabled population. Moreover, we found that participating in leisure activities was more closely associated with stress, whereas participating in social activities was more closely associated with depression. Accordingly, studying the characteristics of different activities, as well as ways to improve the level of participation in beneficial activities, is warranted.

## Figures and Tables

**Table 1:** General characteristics of subjects included for analysis at baseline.

	Total		Stress					Depressive Symptoms			
			No		Yes		*p*-Value	Yes		No	
	N	%	N	%	N	%		N	%	N	%
**Degree of participation in social activities in daily life**							0.1519				
Not at all	107	7.03	43	40.2	64	59.8		23	21.5	84	78.5
Not much	492	32.35	198	40.2	294	59.8		77	15.7	415	84.4
Somewhat	823	54.11	370	45.0	453	55.0		61	7.4	762	92.6
Very much	99	6.51	50	50.5	49	49.5		6	6.1	93	93.9
**Daily average leisure time (weekends)**							0.0002				
4 or fewer hours	590	38.79	219	37.1	371	62.9		72	12.2	518	87.8
5–9 h	710	46.68	330	46.5	380	53.5		72	10.1	638	89.9
10 h or more	221	14.53	112	50.7	109	49.3		23	10.4	198	89.6
**Daily average leisure time (weekdays)**							0.2197				
2 or fewer hours	612	40.24	259	42.3	353	57.7		77	12.6	535	87.4
3–4 h	635	41.75	270	42.5	365	57.5		60	9.5	575	90.6
5 h or more	274	18.01	132	48.2	142	51.8		30	11.0	244	89.1
**Gender**							0.4225				
Male	1142	75.08	503	44.1	639	42.0		121	10.6	1021	89.4
Female	379	24.92	158	41.7	221	58.3		46	12.1	333	87.9
**Age**							0.1753				
15–29	229	15.06	114	49.8	115	50.2		24	10.5	205	89.5
30–39	469	30.83	196	41.8	273	58.2		45	9.6	424	90.4
40–49	499	32.81	205	41.1	294	58.9		60	12.0	439	88.0
50–59	226	14.86	105	46.5	121	53.5		25	11.1	201	88.9
>59	98	6.44	41	41.8	57	58.2		13	13.3	85	86.7
**Residential region**							0.1979				
Metropolitan	321	21.10	139	43.3	182	56.7		45	14.0	276	86.0
Urban	410	26.96	193	47.1	217	52.9		23	5.6	387	94.4
Rural	790	51.94	329	41.7	461	58.4		99	12.5	691	51.0
**Marital status**							0.1296				
Married	823	54.11	340	41.3	483	58.7		74	9.0	749	91.0
Single	520	34.19	244	46.9	276	53.1		59	11.4	461	88.7
Divorced, separated	178	11.7	77	43.3	101	56.7		34	19.1	144	80.9
**Self-rated health**							<.0001				
Poor	425	27.94	130	30.6	295	34.3		79	18.6	346	81.4
Good	1096	72.06	531	48.5	565	51.6		88	8.0	1008	92.0
**Smoking status**							0.0898				
Current smoker	435	28.6	171	39.3	264	60.7		60	13.8	375	86.2
Former smoker	345	22.68	150	43.5	195	56.5		39	11.3	306	88.7
Nothing	741	48.72	340	45.9	401	54.1		68	9.2	673	90.8
**Alcohol consumption**							0.1185				
Drinker	880	57.89	363	41.3	517	58.8		98	11.1	782	88.9
Former drinker	248	16.32	112	45.2	136	54.8		31	12.5	217	87.5
Nothing	392	25.79	185	47.2	207	52.8		38	9.7	354	90.3
**Depressive symptoms/Stress**							<0.0001				
Yes/No	167	10.98	26	15.6	141	84.4		26	3.9	635	96.1
No/Yes	1354	89.02	635	46.9	719	53.1		141	16.4	719	83.6
**Disability grade**							0.6559				
1–3	394	25.9	175	44.4	219	55.6		50	12.7	344	87.3
4–6	1127	74.1	486	43.1	641	56.9		117	10.4	1010	89.6
**Disability type**							0.9457				
Physical disability	866	56.94	377	43.5	489	56.5		85	9.8	781	90.2
Other	655	43.06	284	43.4	371	56.6		82	12.5	573	87.5
**Total**	**1521**	**100**	661	43.5	860	56.5		167	11.0	1354	89.0

**Table 2:** Association between average leisure time and mental health.

	**Stress**				**Depressive Symptoms**			
	***OR***	**95% CI**		***p*-Value**	***OR***	**95% CI**		***p*-Value**
**Daily average leisure time (weekdays)**								
2 or fewer hours	1.461	1.193	1.789	0.000	0.968	0.664	1.411	0.865
3–4 h	1.261	1.050	1.515	0.013	0.911	0.647	1.283	0.595
5 h or more	1.000				1.000			
**Daily average leisure time (weekends)**								
4 or fewer hours	1.176	0.946	1.463	0.145	1.465	0.980	2.191	0.063
5–9 h	1.223	1.007	1.486	0.043	1.030	0.712	1.490	0.877
10 h or more	1.000				1.000			

Adjusted variables: gender, age, residential region, marital status, self-rated health, smoking status, alcohol consumption, depressive symptoms, stress, disability grade, disability type, and year.

**Table 3:** Association between degree of participation in social activities and mental health.

	Stress				Depressive Symptoms			
	*OR*	95% CI		*p*-Value	*OR*	95% CI		*p*-Value
**Degree of participation in social activities in daily life**								
Not at all	1.000				1.000			
Not much	1.070	0.836	1.370	0.589	0.711	0.502	1.005	0.054
Somewhat	1.074	0.839	1.373	0.572	0.356	0.246	0.515	<0.0001
Very much	0.972	0.693	1.364	0.871	0.314	0.156	0.633	0.001

Adjusted variables: gender, age, residential region, marital status, self-rated health, smoking status, alcohol consumption, depressive symptoms, stress, disability grade, disability type, and year.
